# Structure Dependent Electrochemical Behaviors of Hard Carbon Anode Materials Derived from Natural Polymer for Next-Generation Sodium Ion Battery

**DOI:** 10.3390/polym15224373

**Published:** 2023-11-10

**Authors:** Jungpil Kim, Sang-Don Han, Bonwook Koo, Sang-Hyun Lee, Junghoon Yang

**Affiliations:** 1 Carbon & Light Materials Application R&D Group, Korea Institute of Industrial Technology, Jeonju 54853, Republic of Korea; jpkim@kitech.re.kr; 2 Department of Chemistry, Sejong University, Seoul 05006, Republic of Korea; shan@sejong.ac.kr; 3 School of Forestry Sciences and Landscape Architecture, Kyungpook National University, Daegu 41566, Republic of Korea; bkoo@knu.ac.kr; 4 Lignum, Daejeon 34134, Republic of Korea

**Keywords:** natural polymer, lignin, hard carbon, anode, sodium ion battery

## Abstract

Hard carbons are one of the most promising anode materials for next-generation sodium-ion batteries due to their high reversible capacity, long cycle life, and low cost. The advantage in terms of price of hard carbons can be further improved by using cheaper resources such as biomass waste as precursors. Lignin is one of the richest natural bio-polymer in the earth which can be obtained from woods. As the lignin has three-dimensional amorphous polymeric structure, it is considered as good precursor for producing carbonaceous materials under proper carbonization processes for energy storage devices. In this study, structural properties of lignin-derived hard carbons such as interlayer spacing, degree of disorder and surface defects are controlled. Specifically, lignin-derived hard carbons were synthesized at 1000 °C, 1250 °C, and 1500 °C, and it was confirmed that the structure gradually changed from a disordered structure to ordered structure through X-ray diffraction, Raman spectroscopy, and X-ray photoelectron spectroscopy. Hard carbons exhibit sloping regions at high voltage and plateau region at low voltage during the electrochemical processes for sodium ions. As the heat treatment temperature increases, the contribution to the overall reversible capacity of the sloping region decreases and the contribution of the plateau region increases. This trend confirms that it affects reversible capacity, rate-capability, and cycling stability, meaning that an understanding of structural properties and related electrochemical properties is necessary when developing hard carbon as a negative electrode material for sodium ion batteries.

## 1. Introduction

Rapid development in renewable and clean energy generation such as solar and wind energy systems have encouraged the improvements in large-scale energy storage systems (ESS) for their efficient utilization [1,2,3,4]. Although lithium ion batteries (LIBs) are representative rechargeable batteries for mobile electronic devices and electric vehicles because of their high energy density, long-term cycle life, and power density, LIBs are difficult to be used for ESS due to concerns about increase in prices due to the limited reserves and uneven distribution of lithium resources in the earth. As an alternative, sodium ion batteries (SIBs) have been regarded as next-generation rechargeable batteries because of the low cost and abundance of sodium resources [5,6,7]. Also, the similar chemistries in working principles of the SIBs compared to LIBs make it more attractive. However, the different physicochemical properties between lithium ions and sodium ions require the development of suitable electrode materials including both anodes and cathodes for SIBs [8,9,10,11,12,13,14,15,16,17,18,19,20,21,22,23,24,25]. 

One of the well-known phenomena caused by the differences between lithium ion and sodium ion can be found in graphite which is the widely used anode material in LIBs [26]. The reason for the inactivity of graphite toward sodium ion storage is thermodynamically instability of binary graphite intercalation compounds (GICs) formation between graphite and sodium ions [26,27]. Therefore, considerable research is being conducted to find alternative carbonaceous anode materials for use in SIBs [28,29,30,31,32]. Among them, hard carbon, which is a non-graphitizable carbonaceous material has attractive tremendous attention due to its electrochemical capability toward sodium ions. Previous studies reported that hard carbons exhibit high reversible capacity with excellent cycling performances and rate capability based on the controllable inter-layer spacing, surface area, pore structure and crystallinity [33,34,35,36,37]. 

The fact that the hard carbons can be produced from environmentally benign biomass with low cost makes them more attractive. Various types of biomass resources have been used as precursors for producing hard carbons [38,39,40,41,42,43,44]. In general, biomass significantly varies in its composition, including cellulose, hemi-cellulose, and lignin depending on the type. Among the key components of biomass, lignin is a class of complex organic polymers and it supports the tissues of most plants. As lignin is the richest natural biopolymer in the earth, it has potential to be used for synthesizing carbonaceous anode materials [45,46]. Lignin is currently mainly produced as a by-product in the paper and pulp industries, and the growing biofuel generation industry by using biomass is expected to result in the production of huge amounts of lignin. However, lignin is mostly being discarded due to a lack of appropriate methods to use [47,48]. Therefore, the conversion of lignin into hard carbon as an anode material for SIBs could be a suitable strategy to meet the gradually increasing demands of electrode materials in rechargeable markets [49]. In general, it is generally accepted that the three-dimensional amorphous polymeric structure of lignin is mainly composed of three oxidatively coupled 4-hydroxyphenylpropanoid units, differing in their degrees of methoxylation [50]. Despite the complex structural features of lignin, the relatively high carbon content (60–75%) of lignin can provide a high yield for hard carbon products. However, there is a lack of knowledge about how various physicochemical properties of lignin affect the final structure of hard carbon. In this study, we investigate the relationship between the structural properties and electrochemical behaviors of hard carbons as anodes for SIBs. The natural biopolymer (lignin) was used as a precursor for hard carbon synthesis, and the differences in the structural properties were controlled by varying the heat-treatment temperature conditions at 1000 °C, 1250 °C, and 1500 °C. The structural analysis revealed that the relative ratio of graphitic region (or ordered region) in hard carbons increased as the heat-treatment temperature increased. As a result, different contributions to their electrochemical signal for Na^+^ ions insertion and extraction, which are exhibited as a slope region and a plateau region in voltage profiles, were observed. In detail, the development of ordered region in hard carbons results in the increase of capacity contribution from low voltage plateau region. However, it is not proper to improve the kinetics of the hard carbons at high current density condition. Therefore, it is important to control the microstructural properties of hard carbons to improve the reversible capacity, cycling stability, and rate-capability of hard carbon anode for SIB.

## 2. Materials and Methods

### 2.1. Materials Preparation

For the preparation of lignin-derived hard carbons, the lignin precursor was produced in Lignum (Daejeon, Republic of Korea) and used without further purification. 20 g of lignin precursor was carbonized in N_2_ atmosphere at 1000 °C (L-HC-1000), 1250 °C (L-HC-1250) and 1500 °C (L-HC-1500), respectively for 1 h with a ramping rate of 10 °C/min. The yield of the L-HC-1000, L-HC-1250 and L-HC-1500 is 18.5%, 17.1% and 16.8%, respectively.

### 2.2. Material Characterization

The structural properties of hard carbons were analyzed by powder X-ray diffractometer (XRD, Ultima V, Rigaku, Tokyo, Japan) and Raman spectroscope (RAMANtouch, nanophoton Corp, Osaka, Japan). The morphologies of the materials were analyzed using a field-effect scanning electron microscope (FE-SEM, JSM-7100F, JEOL Ltd., Tokyo, Japan). The chemical states of hard carbons were determined by X-ray photoelectron spectroscopy (Thermo/K-Alpha ESCA System, Thermo Fisher Scientific Inc., Waltham, MA, USA) 

### 2.3. Electrochemical Analysis

The electrode of hard carbons was prepared by mixing the active materials (80 wt%), acetylene black (10 wt%), and poly-vinylidenefluoride (PVdF, 10 wt%) in an N-methylpyrrolidone (NMP) solvent. The slurry was coated onto Cu foil using a doctor blade and then dried in a vacuum oven at 100 °C for 5 h. After drying, the electrodes were pressed and then punched into a round shape with diameter of 1.4 cm. The average loading density of active material on the current collector was 3.5 mg cm^−2^. The electrochemical properties of the prepared electrodes were evaluated using CR 2032 coin-type cells that were assembled in an Ar-filled glovebox. Na metal foil was used as a counter and reference electrode, and 1 M NaPF_6_ dissolved in diethylene glycol dimethyl ether (DEGDME) was employed as an electrolyte. The electrochemical performances of the assembled coin-type cells were tested by galvanostatic charge–discharge analysis at room temperature in the voltage range of 2.0–0.01 V (Na/Na^+^) at various current density conditions using battery cycler (WBCS 3000, Wonatech, Seoul, Republic of Korea).

## 3. Results

**Figure 1:**
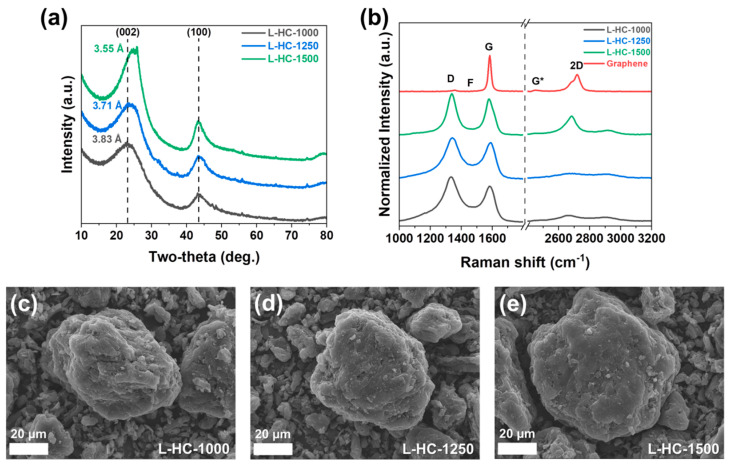
(**a**) XRD patterns of lignin-derived hard carbons. (**b**) Raman spectra of L-HC-1000, L-HC-1250, L-HC-1500, and graphene reference. Morphological observation of lignin-derived hard carbons analyzed by FE-SEM for (**c**) L-HC-1000, (**d**) L-HC-1250 and (**e**) L-HC-1500.

The lignin precursors were carbonized at 1000 °C, 1250 °C, and 1500 °C to synthesize L-HC-1000, L-HC-1250, and L-HC-1500, respectively. The temperature variation was intended to investigate the effects of the carbonization temperature on the structural properties of hard carbons. Initially, the structural characteristics of lignin-derived hard carbons were analyzed using XRD, as shown in Figure 1a. The XRD patterns exhibit two main peaks located at two-thetas of 23–25° and 43.5°, which correspond to the (002) and (100) diffraction planes of graphite. The broad shape of the peaks suggests the amorphous carbon structure of all hard carbons, regardless of carbonization temperatures. The position of the (002) plane peak slightly shifted toward a higher two-theta value with increasing of carbonization temperature, indicating an increase in the ordered region of hard carbon local structure. The average interlayer spacing values of lignin-derived hard carbons are shown in Figure 1a. The detailed microstructures were further analyzed using Raman spectroscopy, as shown in Figure 1b. In the Raman spectra, the following five bands were assigned: (1) the D band (~1355 cm^−1^) originating from defects and/or disordered sp^3^ carbon, (2) the F band (~1430 cm^−1^) originating from C-C stretching modes in 5–6-membered rings; (3) the G band (~1586 cm^−1^) originating from sp^2^ carbon, (4) the G* band (~2445 cm^−1^) originating from turbostratic graphite, and (5) the 2D band (2730 cm^−1^) originating from ordered structures similar to graphite [51,52,53,54]. The spectra were normalized with respect to the G band intensity for quantitative analysis. For L-HC-1000, L-HC-1250, and L-HC-1500 samples, as the heat treatment temperature increased, the intensities of the D and F bands, associated with defects, decreased, while the intensities of the G* and 2D bands increased. The characteristic 2D band, typically observed in the Raman spectrum of graphene reference with minimal defects, is also detected only in the L-HC-1500 sample. This indicates that L-HC-1500 has the most ordered structure similar to graphite among lignin-derived hard carbons. Numeric values for band intensity ratios such as I_D_/I_G_, I_F_/I_G_, and I_2D_/I_G_ are shown in Table 1. Figure 1c–e shows the morphology of particles analyzed by FE-SEM for L-HC-1000, L-HC-1250, and L-HC-1500, respectively. Basically, lignin-derived hard carbons have a spherical particle shape with a particle size range from 10 to 60 µm. It is noticeable that the overall morphological characteristics of hard carbons were not greatly affected by the carbonization temperature.

**Figure 2:**
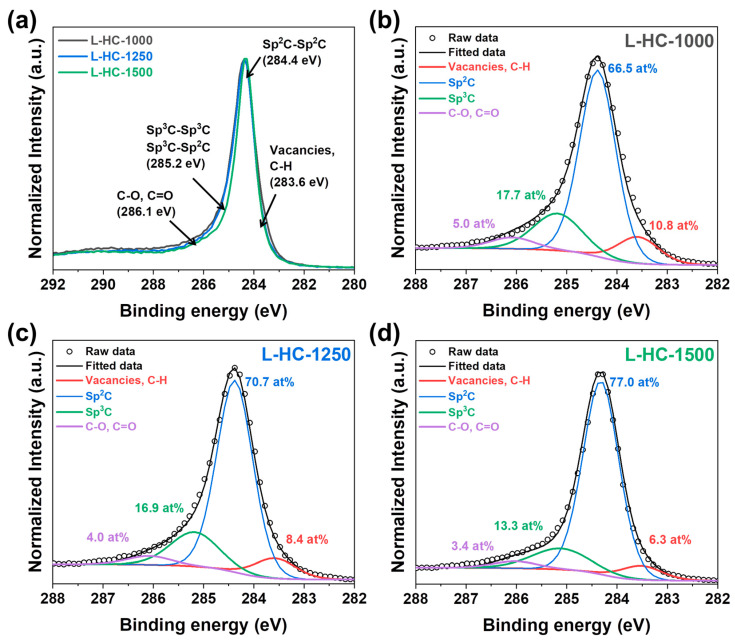
High resolution C1s XPS spectra of lignin-derived hard carbons. (**a**) Comparison of normalized C1s spectra of lignin-derived hard carbons. Peak deconvolution of high resolution C1s spectra of (**b**) L-HC-1000, (**c**) L-HC-1250 and (**d**) L-HC-1500.

The chemical state of carbon atoms of lignin-derived hard carbons with respect to heat-treatment temperature changes were further investigated by XPS analysis as shown in Figure 2. In general, the C1s spectra of carbonaceous materials can be separated by several species including vacancies/C-H (on edges), Sp^2^ carbon, Sp^3^ carbon and C-O/C=O that is located at 283.6 eV, 284.4 eV, 285.2 eV and 286.1 eV, respectively (Figure 2a) [55]. As shown in Figure 2a, the full width at half maximum (FWHM) of the lignin-derived hard carbons decreased with increasing of heat-treatment temperature, indicating lower content of vacancies and/or C-H, sp^3^ carbon and C-O and/or C=O species in the L-HC-1500 than that of L-HC-1000 and L-HC-1500. Those changes in FWHM are related to the gradual structural changes of disorder to order with the increasing of heat-treatment temperature as we already observed from XRD and Raman analysis. The more detailed information is analyzed by the deconvolution of the high resolution C1s XPS spectra as shown in Figure 2b–d and Table 2. As shown, the relative ratio of the peaks related to carbon to carbon (sp^2^ carbon and sp^3^ carbon) for L-HC-1000, L-HC-1250, and L-HC-1500 are 84.2 at %, 87.6 at % and 90.3 at %, respectively. At the same time, it was confirmed that as the heat treatment temperature increases, the ratio of peaks related to vacancy/C-H and C-O/C=O decreases. For vacancy/C-H peak, L-HC-1000, L-HC-1250, and L-HC-1500 contain 10.8 at%, 8.4 at% and 6.3 at%, respectively. Also, the C-O/C=O species decreased in the order of 5.0 at%, 4.0 at%, and 3.4 at% with increasing of heat-treatment temperature in lignin-derived hard carbons. These results imply that the higher carbonization temperatures induce removal of vacancies and oxygen species from the lignin precursor, and then arranging the carbon atoms into more ordered structure. These results are in good agreement with the results mentioned in XRD and Raman analysis.

**Figure 3:**
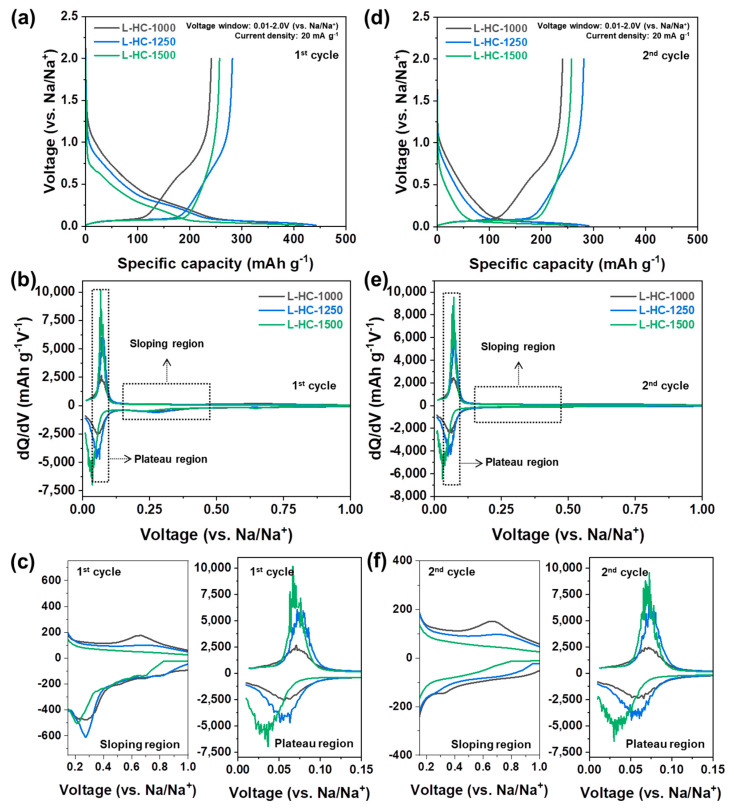
(**a**) Galvanostatic sodiation and desodiation profiles of L-HC-1000, L-HC-1250, and L-HC-1500 in initial cycle and (**b**) corresponding dQ/dV plots. (**c**) Magnified view of the dQ/dV plots in initial cycle for the sloping region and plateau region. (**d**) Galvanostatic sodiation and desodiation profiles of L-HC-1000, L-HC-1250, and L-HC-1500 in second cycle and (**e**) corresponding dQ/dV plots. (**f**) Magnified view of the dQ/dV plots in second cycle for the sloping region and plateau region.

The electrochemical properties of lignin-derived hard carbons as anode materials for NIBs are analyzed to investigate the effect of structural properties on electrochemical behaviors (Figure 3). The galvanostatic charge and discharge profiles of L-HC-1000, L-HC-1250 and L-HC-1500 were analyzed in the voltage window of 0.01–2.0 V (vs. Na/Na^+^, hereafter) at a current density of 20 mA g^−1^. As shown in Figure 3, the electrochemical behaviors of hard carbon can be divided into two major signals: sloping regions (above 0.1 V) and plateau regions (below 0.1 V). In general, the high-voltage sloping capacity is attributed to sodium ion adsorption on surface active sites, including defects, turbostratic disorder sites, and/or chemical functionalities, while the low-voltage plateau capacity is attributed to Na^+^ insertion into the graphitic layers [35,56]. In the initial cycle, L-HC-1000, L-HC-1250, and L-HC-1500 exhibit 388.4 mAh g^−1^, 441.7 mAh g^−1^, and 417.8 mAh g^−1^ of sodiation capacity, respectively. In the subsequent desodiation process, L-HC-1000, L-HC-1250, and L-HC-1500 exhibit 241.3 mAh g^−1^, 281.6 mAh g^−1^, and 256.9 mAh g^−1^ of initial de-sodiation capacity, respectively. It should be noted that the L-HC-1250, which was treated at the intermediate heat-treatment temperature, shows the highest reversible capacity, indicating that the structural characteristics clearly affects the electrochemical behaviors. The initial Coulombic efficiencies of L-HC-1000, L-HC-1250, and L-1500 are 62.12%, 63.75%, and 61.48%, respectively. Generally, the low initial Coulombic efficiency of carbonaceous anode materials originates from electrolyte decomposition and SEI formation [57]. The detailed electrochemical behaviors are further analyzed by the differential analysis (dQ/dV) plots, as shown in Figure 3b. For better comparison, the differential plots for the sloping region and plateau region are magnified, respectively (Figure 3c). In the sloping region, the area of the differential plot increase in the order of L-HC-1000, L-HC-1250, and L-HC-1500. On the contrary, the intensity of the differential signal in the plateau region increases in the order of L-HC-1500, L-HC-1250, and L-HC-1000. Furthermore, it is noticed that the oxidation and reduction peaks of L-HC-1500 observed in the plateau region are located at relatively lower voltages compared with L-HC-1000 and L-HC-1250. These results indicate that the L-HC-1000 and L-HC-1500 have completely different capacity contributions from sloping regions and plateau regions, while the L-HC-1250 exhibits intermediate characteristics. The galvanostatic charge and discharge profiles and corresponding differential plots are shown in Figure 3d–f. The electrochemical behaviors of lignin-derived hard carbons obtained at the second cycle are similar to those of the initial cycle, except for the improved Coulombic efficiency value. In the second cycle, L-HC-1000, L-HC-1250, and L-HC-1500 exhibit 248.8 mAh g^−1^, 290.3 mAh g^−1^, and 269.0 mAh g^−1^ of sodiation capacity, respectively. The significantly reduced sodiation capacity compared to the initial cycle is due to suppressed electrolyte decomposition by the already-formed SEI layer in the initial cycle. Desodiation capacities of 240.5 mAh g^−1^, 281.2 mAh g^−1^ and 257.7 mAh g^−1^ are obtained for L-HC-1000, L-HC-1250 and L-HC-1500. Based on the measured capacity, the Coulombic efficiencies of L-HC-1000, L-HC-1250 and L-1500 at the second cycle are 96.66%, 96.86%, and 95.79%, respectively. The dQ/dV plots obtained at the second cycle shown in Figure 3e,f show that the tendency for the electrochemical behaviors of lignin-derived hard carbons is still similar to that of the initial cycle. 

**Figure 4:**
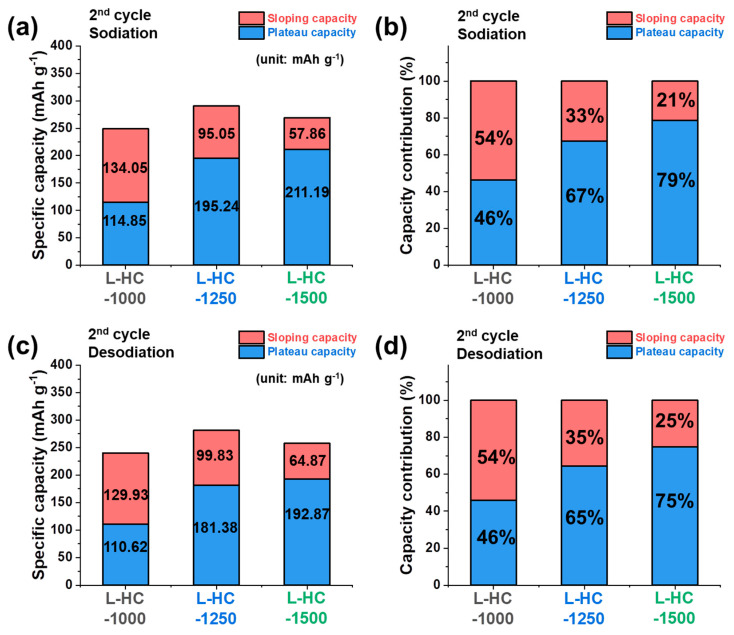
Capacity contribution of L-HC-1000, L-HC-1250 and L-HC-1500 according to voltage regions in the second cycle. (**a**) Separation of sloping capacity and plateau capacity and (**b**) corresponding capacity contribution during second sodiation process. (**c**) Separation of sloping capacity and plateau capacity and (**d**) corresponding capacity contribution during second desodiation process.

In order to further understand the electrochemical behaviors of lignin-derived hard carbons prepared at different heat treatment temperatures, the capacity values were separated according to the voltage range, as shown in Figure 4. Because the initial sodiation process is accompanied by an electrolyte decomposition reaction, we would focus more on the explanation of capacity contribution analysis for the second cycle in this part. In the second cycle, the sloping capacity of 134.05 mAh g^−1^, 95.05 mAh g^−1^, and 57.86 mAh g^−1^ and the plateau capacity of 114.85 mAh g^−1^, 195.24 mAh g^−1^, and 211.19 mAh g^−1^ were obtained for L-HC-1000, L-HC1250, and L-HC-1500, respectively, in the sodiation process (Figure 4a). The relative ratio of the capacity contribution according to voltage regions is shown in Figure 4b. The relative ratio of sloping capacity for overall capacity of L-HC-1000, L-HC-1250, and L-HC-1500 is 54%, 33%, and 21%, respectively (Figure 4b). In other words, the contribution of plateau capacity increases in the order of 46%, 67%, and 79% for L-HC-1000, L-HC-1250, and L-HC-1500 as the heat-treatment temperature for hard carbon synthesis increases. These results match well with the expectations we had from the differential plot analysis. As mentioned in the previous report, the different electrochemical signals observed by the sloping region and plateau region are related to Na^+^ ion uptakes on the surface active sites of hard carbons and interlayer space between ordered graphitic layers of hard carbons, respectively [35]. Therefore, it is reasonable that the L-HC-1500, which has the most ordered structures among all lignin-derived hard carbons, has the highest capacity contribution from the plateau region. The tendency of the capacity contribution analysis observed at the second desodiation capacity is similar to that of the sodiation process, as shown in Figure 4c,d. From the analysis results, it is confirmed that the highest reversible capacity of L-HC-1250 among all lignin-derived hard carbons is contributed by both sloping capacity and plateau capacity at the same time.

**Figure 5:**
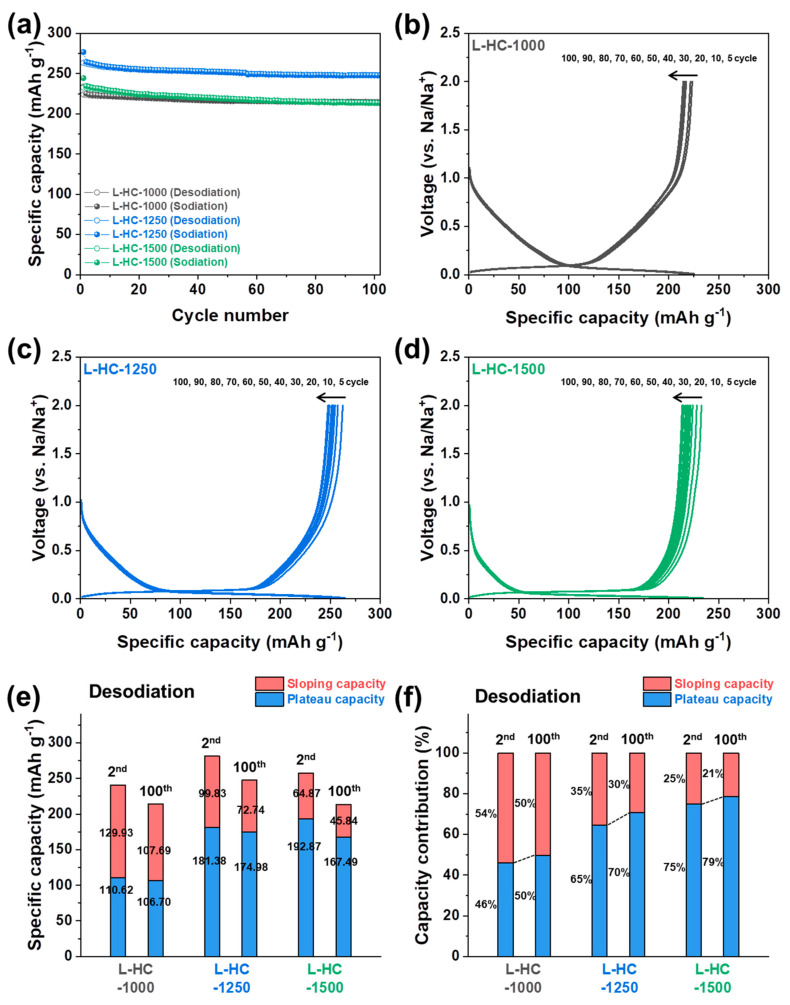
(**a**) Cycling stability plot (at current density of 20 mA g^−1^) of L-HC-1000, L-HC-1250 and L-HC-1500 up to 100 cycles. Galvanostatic sodiation and desodiation profiles of (**b**) L-HC-1000, (**c**) L-HC-1250 and (**d**) L-HC-1500 obtained at different cycle number s. (**e**) Comparison of separated desodiation capacity value according to voltage region and (**f**) corresponding capacity contribution between 2nd and 100th cycle.

The repetitive cycling stability test results of the lignin-derived hard carbons up to 100 cycles are measured at a current density of 20 mA g^−1^ as shown in Figure 5. The observed desodiation capacities of L-HC-1000, L-HC-1250, and L-HC-1500 after 100 cycles are 214.40 mAh g^−1^, 247.72 mAh g^−1^, and 213.33 mAh g^−1^, respectively. The values correspond to 95.7%, 94.1%, and 91.6% of the initial desodiation capacity, indicating good reversibility of lignin-derived hard carbons toward repetitive electrochemical processes. It is noticeable that the L-HC-1500, which mainly exhibits plateau capacity, exhibits the lowest capacity retention among all lignin-derived hard carbons. In other words, L-HC-1000, which shows the most capacity in a sloping region, has the highest stability during repetitive charge and discharge processes. To have more insights into the capacity fading of lignin-derived hard carbons according to different structural properties, the galvanostatic sodiation and desodiation profiles of L-HC-1000, L-HC-1250, and L-HC-1500 obtained at different cycle numbers are plotted in Figure 5b–d, respectively. As shown, the voltage profile shapes of the hard carbons are well maintained during repetitive cycling, indicating there were no clear changes in electrochemical behaviors regardless of heat-treatment temperature. To clarify the origin of capacity fading, the specific capacity according to voltage region and corresponding capacity contribution changes between the second cycle and the 100th cycle are compared in Figure 5e,f. In the case of L-HC-1000, the sloping capacity decreased from 129.93 mAh g^−1^ to 107.69 mAh g^−1^, and the plateau capacity decreased from 110.62 mAh g^−1^ to 106.70 mAh g^−1^. It is noticeable that most capacity fading is originated from sloping capacity rather than plateau capacity. For L-HC-1250, the sloping capacity decreased from 99.83 mAh g^−1^ to 72.74 mAh g^−1^, and the plateau capacity decreased from 181.38 mAh g^−1^ to 174.98 mAh g^−1^. These results indicate that the sloping capacity decreased by 22.24 mAh g^−1^ and the plateau capacity decreased by 6.4 mAh g^−1^ after 100 cycles. Similar to L-HC-1000, most of the capacity fading for L-HC-1250 is due to the degradation of sloping capacity rather than plateau capacity. Lastly, L-HC-1500 shows a sloping capacity decrease from 64.87 mAh g^−1^ to 45.84 mAh g^−1^, and a plateau capacity decrease from 192.87 mAh g^−1^ to 167.49 mAh g^−1^. Unlike L-HC-1000 and L-HC-1250, L-HC-1500 shows capacity-fading behaviors from both the sloping region and the plateau region at the same time. These results indicate that the capacity fading of the hard carbons does not depend on specific sodium ion storage behavior but rather is caused by structural differences.

**Figure 6:**
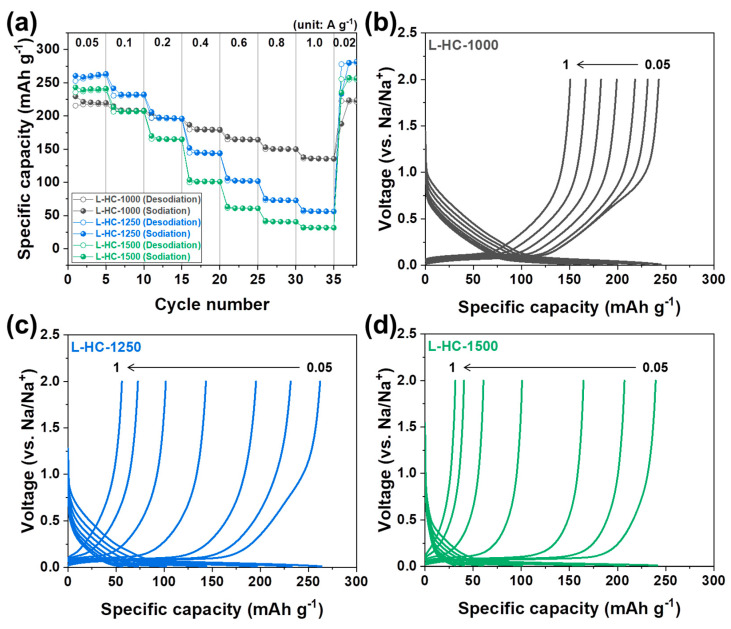
(**a**) Rate capability test results of L-HC-1000, L-HC-1250 and L-HC-1500 obtained at current density of 0.05, 0.1, 0.2, 0.4, 0.6, 0.8 and 1.0 A g^−1^. Galvanostatic sodiation and desodiation profiles obtained from different current density condition of (**b**) L-HC-1000, (**c**) L-HC-1250 and (**d**) L-HC-1500.

The effects of structural differences in hard carbon anodes on rate capability are analyzed in Figure 6. As shown in Figure 6a, the rate capability tests were conducted by varying the current density value from 0.05 A g^−1^ to 1.0 A g^−1^. The reversible capacities of the samples under current densities of 0.05 A g^−1^, 0.1 A g^−1^, 0.2 A g^−1^, 0.4 A g^−1^, 0.8 A g^−1^, and 1.0 A g^−1^ are 215, 207, 196, 179, 164, 150, and 135 mAh g^−1^ for L-HC-1000, 252, 230, 197, 144, 102, 73, and 56 mAh g^−1^ for L-HC-1250, and 235, 206, 165, 99, 61, 40, and 31 mAh g^−1^ for L-HC-1500, respectively. As clearly observed, L-HC-1000 exhibits the highest reversible capacity value at the highest current density condition. The capacity retention values obtained by dividing the capacity value observed at 1.0 A g^−1^ by the value observed at 0.05 A g^−1^ are 62.7%, 22.2%, and 17% for L-HC-1000, L-HC-1250, and L-HC-1500, respectively. To clarify the electrochemical behaviors during rate-capability tests, the galvanostatic sodiation and desodiation profiles obtained at different current density conditions are plotted in Figure 6b–d for L-HC-1000, L-HC-1250, and L-HC-1500, respectively. An interesting phenomenon in the rate capability tests is that the L-HC-1000 exhibits well-maintained original voltage profiles, even at higher current density conditions. Meanwhile, at a current density of 1.0 A g^−1^, L-HC-1250, and L-HC-1500 almost lost their electrochemical contribution from the low-voltage plateau region. Considering that the origin of the high reversible capacity of L-HC-1250 and L-HC-1500 is contributed by the higher plateau capacity rather than the sloping capacity at low current density conditions, the result can be understood as the kinetically hindered plateau capacity contribution under higher current density conditions. As mentioned, the low-voltage plateau region is exhibited by inserting the Na^+^ ions into the well-ordered regions of hard carbons. Considering that crystalline graphite is not capable of storing Na^+^ ions due to the thermodynamically unstable Na^+^-graphite binary compound, it is assumed that the well-ordered regions of L-HC-1250 and L-HC-1500 are not utilized for Na^+^ ion uptake at high current density conditions. However, the fact that the L-HC-1000 still maintains plateau capacity is not easily explained if the storage of sodium ions is simply defined as a structurally dependent phenomenon. If the sodium ion storage behavior is simply correlated to the structure, the L-HC-1000 should also exhibit the most electrochemical activity in a sloping region. Therefore, we need to interpret the rate capability test results as the sequential ion diffusion process from disordered region to ordered region. At low current density conditions, sodium ions can be stored in the ordered carbon structure for a sufficient time. In that case, both L-HC-1000, which has the most disordered structure, and L-HC-1500, which has the most ordered structure were able to store sodium ions from the sloping region by adsorption mechanism and the plateau region by insertion mechanism at the same time. However, different environments for ion storage behaviors appeared if the current density increased. In the case of L-HC-1000, it provides a sufficient disordered region for sodium ion transfer through a sloping region, and then the sodium ions can sequentially be moved to the ordered region. However, the L-HC-1500 could not utilize its well-developed ordered region because it has an insufficiently disordered region for sodium ions to initially be transferred. The lignin-derived hard carbon anodes show good electrochemical properties when compared with previously reported studies [58,59,60,61]. However, the electrochemical properties can be further improved by fine-tuning the microstructural properties, such as domain size and arrangement of disorder and/or order regions.

## 4. Conclusions

In summary, we have synthesized hard carbon anode materials by using a naturally abundant polymer called lignin through a controlled heat-treatment temperature. The lignin-derived hard carbons were prepared at different heat-treatment temperatures of 1000 °C, 1250 °C, and 1500 °C. It was confirmed that the structural characteristics of hard carbons became more ordered as the heat treatment temperature increased. We have studied the correlation between the structural properties of lignin-derived hard carbons and their electrochemical properties to gain insights into structure dependent sodium ion storage behaviors. We have observed that hard carbons have two major electrochemical behaviors: the sloping region by sodium ion adsorption on surface active sites, including defects sites, turbostratic disorder sites and/or chemical functionalities, and the plateau region at sodium ion insertion into the graphitic layers. With an increase in heat-treatment temperature, the sloping capacity decreased and the plateau capacity increased. As a result, the L-HC-1250, which was prepared at an intermediate temperature, exhibits the highest reversible capacity of ~280 mAh g^−1^ measured at a current density of 20 mA g^−1^, gaining benefit from both sloping capacity and plateau capacity at the same time. During repetitive cycling tests, L-HC-1500 exhibits the lowest capacity retention by losing electrochemical behaviors from both sloping capacity and plateau capacity. This might be related to the rigid structural properties of L-HC-1500. Also, in terms of rate capability, L-HC-1000 shows the highest reversible capacity value with increasing current density. At a current density of 1.0 A g^−1^, L-HC-1000 exhibits a specific capacity of 135 mAh g^−1^, while L-HC-1250 and L-HC-1500 show 56 and 31 mAh g^−1^, respectively. Considering that L-HC-1000 maintains both a sloping region and a plateau region, we would propose a sequential sodium ion diffusion model from disordered region to ordered region. Overall, it is necessary to design the microstructure of hard carbon anodes to improve the electrochemical properties that is dependent on the structure.

## Figures and Tables

**Table 1:** Numeric values analyzed from Raman spectra of L-HC-1000, L-HC-1250, L-HC-1500 and graphene reference.

Sample	I_F_/I_G_	I_D_/I_G_	I_2D_/I_G_
L-HC-1000	0.42	1.26	0.19
L-HC-1250	0.38	1.13	0.15
L-HC-1500	0.17	1.13	0.53
Graphene reference	0.01	0.04	0.47

**Table 2:** Numeric values analyzed from XPS spectra of L-HC-1000, L-HC-1250, L-HC-1500.

Sample	Vacancy, C-H	Sp^2^C	Sp^3^C	C-O/C=O	Total
L-HC-1000	10.8	66.5	17.7	5.0	100
L-HC-1250	8.4	70.7	16.9	4.0	100
L-HC-1500	6.3	77.0	13.3	3.4	100

## Data Availability

The data presented in this study are available upon request from the corresponding authors.
